# Rare Stochastic Expression of O6-Methylguanine- DNA Methyltransferase (MGMT) in MGMT-Negative Melanoma Cells Determines Immediate Emergence of Drug-Resistant Populations upon Treatment with Temozolomide In Vitro and In Vivo

**DOI:** 10.3390/cancers10100362

**Published:** 2018-09-28

**Authors:** Thomas C. Chen, Nymph Chan, Radu O. Minea, Hannah Hartman, Florence M. Hofman, Axel H. Schönthal

**Affiliations:** 1 Department of Neurosurgery, Keck School of Medicine, University of Southern California, Los Angeles, CA 90089, USA; tcchen@usc.edu (T.C.C.); nymphcha@usc.edu (N.C.); minea@usc.edu (R.O.M.); 2 Department of Pathology, Keck School of Medicine, University of Southern California, Los Angeles, CA 90089, USA; hofman@usc.edu; 3 Department of Molecular Microbiology & Immunology, Keck School of Medicine, University of Southern California, Los Angeles, CA 90089, USA; hlhartma@usc.edu

**Keywords:** O6-methylguanine-DNA methyltransferase, MGMT, chemoresistance

## Abstract

The chemotherapeutic agent temozolomide (TMZ) kills tumor cells preferentially via alkylation of the O6-position of guanine. However, cells that express the DNA repair enzyme O6-methylguanine-DNA methyltransferase (MGMT), or harbor deficient DNA mismatch repair (MMR) function, are profoundly resistant to this drug. TMZ is in clinical use for melanoma, but objective response rates are low, even when TMZ is combined with O6-benzylguanine (O6BG), a potent MGMT inhibitor. We used in vitro and in vivo models of melanoma to characterize the early events leading to cellular TMZ resistance. Melanoma cell lines were exposed to a single treatment with TMZ, at physiologically relevant concentrations, in the absence or presence of O6BG. Surviving clones and mass cultures were analyzed by Western blot, colony formation assays, and DNA methylation studies. Mice with melanoma xenografts received TMZ treatment, and tumor tissue was analyzed by immunohistochemistry. We found that MGMT-negative melanoma cell cultures, before any drug treatment, already harbored a small fraction of MGMT-positive cells, which survived TMZ treatment and promptly became the dominant cell type within the surviving population. The MGMT-negative status in individual cells was not stable, as clonal selection of MGMT-negative cells again resulted in a mixed population harboring MGMT-positive, TMZ-resistant cells. Blocking the survival advantage of MGMT via the addition of O6BG still resulted in surviving clones, although at much lower frequency and independent of MGMT, and the resistance mechanism of these clones was based on a common lack of expression of MSH6, a key MMR enzyme. TMZ treatment of mice implanted with MGMT-negative melanoma cells resulted in effective tumor growth delay, but eventually tumor growth resumed, with tumor tissue having become MGMT positive. Altogether, these data reveal stochastic expression of MGMT as a pre-existing, key determinant of TMZ resistance in melanoma cell lines. Although MGMT activity can effectively be eliminated by pharmacologic intervention with O6BG, additional layers of TMZ resistance, although considerably rarer, are present as well and minimize the cytotoxic impact of TMZ/O6BG combination treatment. Our results provide rational explanations regarding clinical observations, where the TMZ/O6BG regimen has yielded mostly disappointing outcomes in melanoma patients.

## 1. Introduction

Resistance to chemotherapeutic agents is a major obstacle preventing successful treatment of cancer. The exact origin of drug resistance is still debated, with two major models put forward. One model poses that so-called drug-tolerant cells (DTCs) just sufficiently adjust to drug exposure to provide them with enough time to acquire effective mechanisms of resistance. The other model implies that drug-resistant subclones already exist before drug exposure, and then are selected for by treatment [1,2]. There are examples to support the existence of both of these processes, although their occurrence may vary depending on the tumor type and specifics of therapeutic intervention, such as drug dosage, exposure time, treatment cycles, and mechanism of drug action.

Studies that investigate the features of drug-resistant tumor cells in vitro frequently employ long-term culture of tumor cells, where slowly increasing concentrations of a given drug are repeatedly added over the course of weeks or many months [3,4,5,6,7]. In the in vivo situation, or in patient tissues, as well, when before-and-after specimens are being compared, the samples usually differ by many cycles of drug treatment [8,9,10,11]. These extended treatment schedules make it difficult to investigate the early parameters that determine drug resistance. In particular, they are not well suited to addressing the question as to whether drug resistance predated the onset of treatment, or whether tumor cells adapted slowly over time, perhaps aided by drug-induced mutations [12,13,14]. To avoid some of these limitations, we designed an in vitro study where cultured tumor cells were exposed to only a single round of chemotherapeutic agent at physiologically relevant concentrations, followed by analysis of surviving cells.

We studied temozolomide (TMZ), an alkylating agent whose cell killing mechanism is very well characterized [15]. TMZ is a pro-drug and an imidazotetrazine derivative of dacarbazine. Under neutral pH and in aqueous conditions, it spontaneously decarboxylates to generate 5-(3-methyltriazen-1-yl)imidazole-4-carboxamide (MTIC), which further degrades to 4-amino-5-imidazole-carboxamide (AIC), plus a highly reactive methyldiazonium ion that acts as the DNA methylating species [16]. While up to a dozen targets within the DNA strand have been identified, it is methylation of the O6 position of guanine that represents the key toxic insult [17]. However, methylated O6-guanine can efficiently be repaired by the DNA repair enzyme O6-methylguanine-DNA methyltransferase (MGMT), which is the reason MGMT-overexpressing cells are profoundly resistant to TMZ [18,19,20,21]. On the other hand, O6-benzylguanine (O6BG), a highly effective inhibitor of MGMT [22], restores sensitivity of MGMT-positive cells to killing by TMZ [23,24].

While TMZ’s approved indication is malignant glioma, it has also been used for some other cancer types, although generally with less convincing therapeutic benefit. One such example is advanced melanoma. Melanoma incidence is increasing and, despite recent therapeutic advances, the prognosis for patients with metastatic disease remains poor [25]. Traditionally, first-line treatment of metastatic melanoma has included the methylating agent dacarbazine or its oral analog TMZ [26]. Newer agents have been approved recently, such as B-RAF inhibitors vemurafenib and dabrafenib, immune checkpoint inhibitory monoclonal antibodies ipilimumab and nivolumab, MEK (mitogen-activated protein kinase kinase) inhibitor trametinib, and others (see details in review [27]). However, therapeutic responses to these new drugs frequently are short-lived, and many patients with advanced melanoma do not obtain long-lasting clinical benefit. In view of these limitations, and despite its limited clinical efficacy, TMZ has remained an important part of current treatment regimens for patients with metastatic melanoma [28].

As compared to treatment of newly diagnosed GBM, treatment of advanced melanoma with TMZ presents with less success and lower response rates [29,30]. Although a significant correlation of MGMT expression and melanoma resistance to temozolomide has been recognized [18,31,32], clinical trials with added MGMT inhibitors (O6BG or lomeguatrib), were unable to demonstrate substantial improvement upon TMZ’s mediocre therapeutic activity [33,34]. These latter results were disappointing, because in the experimental setting TMZ combined with O6BG has proven very effective in cell culture and animal models [23,35,36]. We therefore designed an approach using well-characterized human melanoma cell lines to address the question as to why melanoma seems to respond to TMZ so relatively poorly, and why the addition of O6BG does not make a dramatic difference. We avoided long-term, repeated drug treatments, but instead performed the majority of our experiments with a single exposure to TMZ, primarily at physiologically relevant concentrations, followed by the analysis of surviving cells.

## 2. Materials and Methods

### 2.1. Pharmacological Agents

TMZ was obtained from the pharmacy at the University of Southern California (USC) or was purchased from Sigma Aldrich (St. Louis, MO, USA) and dissolved in DMSO (Santa Cruz Biotechnology, Dallas, TX, USA) to a concentration of 100 mM. O6-BG (Santa Cruz Biotechnology) was dissolved in DMSO to a concentration of 50 mM. 5-Aza-deoxycytidine (Cayman Chemical, Ann Arbor, MI, USA) was dissolved in DMSO to 100 mM. In all cases of cell treatment, the final DMSO concentration in the culture medium never exceeded 1%, and was much lower in most cases. Stock solutions were stored at −80 °C.

### 2.2. Cell Lines

Human melanoma cell lines A2058, A375 and M24 were propagated in DMEM supplemented with 10% fetal bovine serum (FBS), 100 U/mL penicillin, and 0.1 mg/mL streptomycin in a humidified incubator at 37 °C and a 5% CO_2_ atmosphere. Human melanoma cell line CaCl 74-36 was propagated under similar conditions, except that RPMI was used instead of DMEM. All cell culture reagents were provided by the Cell Culture Core Lab of the USC/Norris Comprehensive Cancer Center and prepared with raw materials from Cellgro/MediaTech (Manassas, VA, USA); FBS was obtained from Omega Scientific (Tarzana, CA, USA) and from X&Y Cell Culture (Kansas City, MO, USA). Initially, experiments were performed with cell lines that were kindly provided by the laboratories of Alan Epstein (USC) [37], Yves DeClerck (USC) [38], and Ali Jazirehi (UCLA) [39]. Subsequently, A2058 and A375 cells were purchased from the American Tissue Culture Collection (Manassas, VA, USA), and these cells were passaged for less than 6 months after receipt, thus representing authenticated cells. All experiments shown in this report used the authenticated versions of A2058 and A375; no differences were noted when results were compared to earlier results obtained with non-authenticated versions of these two cell lines.

### 2.3. Colony Formation Assay

Depending on the cell line and plating efficiency, 400–600 cells were seeded into each well of a 6-well plate and treated as described in detail previously [40]. In the case of co-treatment with O6BG, 15 µM O6BG was added 30–60 min before the addition of TMZ. Two days after the start of drug treatment, the medium was removed and replaced with drug-free medium, except that O6BG was added again. After 12–14 days, formed colonies (groups of >50 cells) were stained with methylene blue and counted. CFAs were generally set up in triplicate, and in most cases were independently repeated once or twice.

### 2.4. Generating Mass Cultures of TMZ-Treated Cells

One hundred thousand cells were seeded into each well of a 6-well culture plate. The next day, increasing concentrations of TMZ were added to the different wells, while one well received vehicle only. After 48 h, medium was replaced with fresh medium. Thereafter, cells were left to recover from drug treatment. Whenever a respective well reached near-confluency, cells were trypsinized and transferred to a 10-cm plate. Reaching near-confluency took longer for cells treated at higher drug concentrations, because initial drug toxicity was greater and the cell population needed more time for recovery. After reaching near-confluency in the 10-cm plate, cells were harvested, lysed, and analyzed by Western blot.

### 2.5. Treatment with 5-Azacytidine

A2058 cells were repeatedly exposed to 50 µM 5-AzaC for 48–72 h. After each treatment, the drug-laced medium was removed and the cells were allowed to recover in drug-free medium. After approximately one week of recovery, the cell culture was transferred onto 2 plates. Cells on one of these plates were harvested for WB analysis, whereas cells on the other plate received the next dose of 5-AzaC. This cycle was repeated 4 times, thus generating cell lysates from cells that had received 1, 2, 3 or 4 rounds of drug treatment.

### 2.6. Immunoblots

Total cell lysates were analyzed by Western blot analysis as described earlier [41]. We used the following primary antibodies. For the detection of MGMT, we used a polyclonal antibody (#2739) from Cell Signaling Technology (Beverly, MA, USA) and a monoclonal antibody (ab39253) from Abcam (Cambridge, MA, USA). Anti-MSH2 (#2017) and anti-MSH6 (#5424) antibodies were from Cell Signaling. Human TRA-1-85 antibody (MAB3195) was from R&D Systems (Minneapolis, MN, USA) and beta-actin antibody (sc-47778) was from Santa Cruz Biotechnology (Santa Cruz, CA, USA). Horseradish peroxidase-antibody conjugates (i.e., secondary antibodies) were obtained from Jackson ImmunoResearch Laboratories (West Grove, PA, USA). All antibodies were used according to the suppliers’ recommendations. All immunoblots were repeated at least once to confirm the results. Band intensities of MGMT were quantified using ImageJ, normalized relative to the respective actin control bands, and expressed in relation to the strongest reference band.

### 2.7. MethyLight Analysis

MGMT promoter methylation status was analyzed by standard MethyLight assay [42], using genomic DNA extracted from A2058 cells and derived clones. The procedure was performed by the USC Pathology Core lab. The primer/probe combination to determine methylated MGMT promoter sequences was the same as the one that is commonly used to analyze MGMT methylation status in glioblastoma patients [43].

### 2.8. Animal Model

All animal protocols were approved by the Institutional Animal Care and Use Committee (IACUC) of USC on 27th July 2016, #20038, and all rules and regulations were followed during experimentation on animals. Athymic mice (Harlan, Inc., Indianapolis, IN, USA) were implanted with 4 × 10^6^ cells into the right flank. About two weeks later, once palpable tumors had developed, animals were assigned to different treatment groups. The control group received vehicle only (45% glycerol, 45% ethanol, 10% DMSO), whereas the treatment group received TMZ dissolved in vehicle. Administration was by gavage.

### 2.9. Tissue Analysis

Upon euthanasia of animals, tumor tissues were collected and stored frozen or fixed in formalin. For the detection of MGMT protein by immunohistochemistry, tumor tissues were processed as described earlier [44]. The primary antibody was mouse monoclonal anti-human MGMT from Abcam (cat #ab39253). The secondary antibody was biotinylated horse anti-mouse IgG (Vector Laboratories, Burlingame, CA, USA), followed by incubation with the Vectastain Elite avidin-biotin-peroxidase complex kit (Vector Laboratories). Positive staining was visualized with amino-ethylcarbazole substrate (red). Hematoxylin was used as the counterstain to mark nuclei (blue).

### 2.10. Statistical Analysis

All parametric data were analyzed using the Student *t*-test to calculate the significance values. A probability value (*p*) < 0.05 was considered statistically significant.

## 3. Results

### 3.1. Sensitivity to TMZ Correlates with MGMT Protein Levels

**Figure 1:**
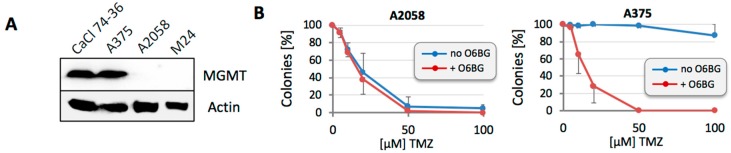
MGMT (O6-methylguanine-DNA methyltransferase) levels and protection from TMZ (temozolomide) toxicity. (**A**) Protein lysates were prepared from four different melanoma cell lines and MGMT protein levels were analyzed by Western blot. Actin was used as the loading control. (**B**) MGMT-negative 2058 cells and MGMT-positive A375 cells were treated with increasing concentrations of TMZ in the absence or presence of 15 µM O6BG. After 12–14 days, emerging colonies were counted. Number of colonies from untreated cells was set at 100% (*n* ≥ 5, ±SE).

For our study, we chose two MGMT-positive (A375 and CaCl 74-36) and two MGMT-negative lines (A2058 and M24) (Figure 1A). As expected, MGMT expression levels in these lines were closely aligned with sensitivity to TMZ, when tested in colony formation assays (CFAs): the IC50 (concentration of drug that reduces colony formation by 50%) was in the range of 10–20 µM for MGMT-negative cells, and well above 100 µM for MGMT-positive cells. Exemplary results are shown in Figure 1B (and similar results were obtained for CaCl 74-36 and M24). To put these data in perspective: achievable peak plasma concentrations of TMZ in patients are 40–50 µM [45,46]. Thus, IC50 values that are greater than 100 µM, as observed for our MGMT-positive cells, clearly indicate profound TMZ resistance. The molecular basis for this resistance could be validated with O6BG, because inclusion of this MGMT inhibitor exquisitely sensitized MGMT-positive A375 cells to TMZ, but had no effect in MGMT-negative A2058 cells (Figure 1B). Essentially identical results were obtained with CaCl 74-36 and M24 cells (not included in the figure).

### 3.2. MGMT-Negative, but Not MGMT-Positive, Cell Populations Adjust to TMZ Treatment

It is generally observed that increasing drug concentrations lead to correspondingly decreasing numbers of emerging colonies in CFAs. However, it is oftentimes unclear why some cells withstand much higher drug concentrations and continue to proliferate. We therefore selected cells that had survived a single round of high-dose drug treatment and investigated the potential basis for their survival. This was done both with MGMT-positive and with MGMT-negative cells.

**Figure 2:**
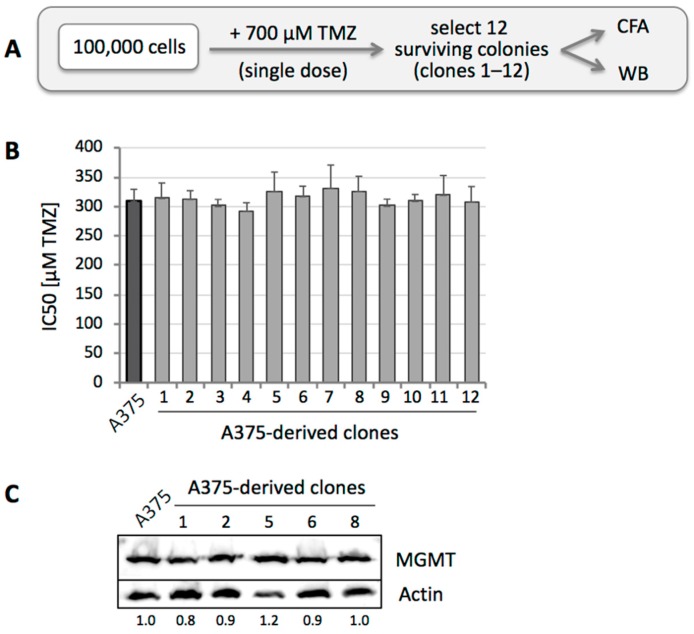
Selection of A375 clones after high-dose TMZ treatment. (**A**) Treatment schedule was as follows: 10^5^ A375 cells (MGMT positive) were seeded into a 10-cm dish and treated with a single dose of 700 µM TMZ for 48 h. Two weeks later, 12 surviving clones (numbered 1 through 12) were isolated for further analysis by WB and CFA. (**B**) All twelve clones were subjected to CFA with increasing concentrations of TMZ. Shown is the average IC50 of each clone, in comparison to parental A375 cells (left bar in black) (*n* = 2–5, ±SE). (**C**) Five of these cell clones were lysed, and MGMT protein levels were analyzed by WB with actin as the loading control. Left lane shows lysate from parental A375 cells. Numbers under the blot indicate quantification of MGMT bands, with reference to the actin signal, and relative to A375 cells (left lane, set at 1.0).

In the case of MGMT-positive A375 cells, the IC50 of TMZ treatment was about 300 µM, yet even at higher concentrations, there were small numbers of survivors. We therefore treated these cells with a single dose of 700 µM TMZ, representing IC99.9. From about 100,000 treated cells, 100 colonies emerged, and 12 individual clones were isolated for further analysis (Figure 2A). We performed CFAs for all 12 of these clones and determined that their IC50s were in a fairly narrow range around 300 µM, i.e., there was no significant change as compared to the non-drug treated parental A375 cells (Figure 2B). Also, when MGMT protein levels were analyzed, no difference was observed between parental cells and individual clones (Figure 2C). Several of these clones were subjected to treatment with TMZ in the presence of O6BG, which caused sensitization to TMZ, confirming that their resistance mechanism was based on MGMT, similar to the parental cells (see Figure 1B). Thus, even though these clones represented the 0.1% fraction of A375 cells able to survive high-dose (700 µM) TMZ, their average drug sensitivity still mirrored that of the 99.9% cells that did not survive drug treatment. In essence, a super-resistant subpopulation could not be established with single drug treatment, and the basis for heterogeneous A375 cell survival at very high drug dosages remains to be established.

**Figure 3:**
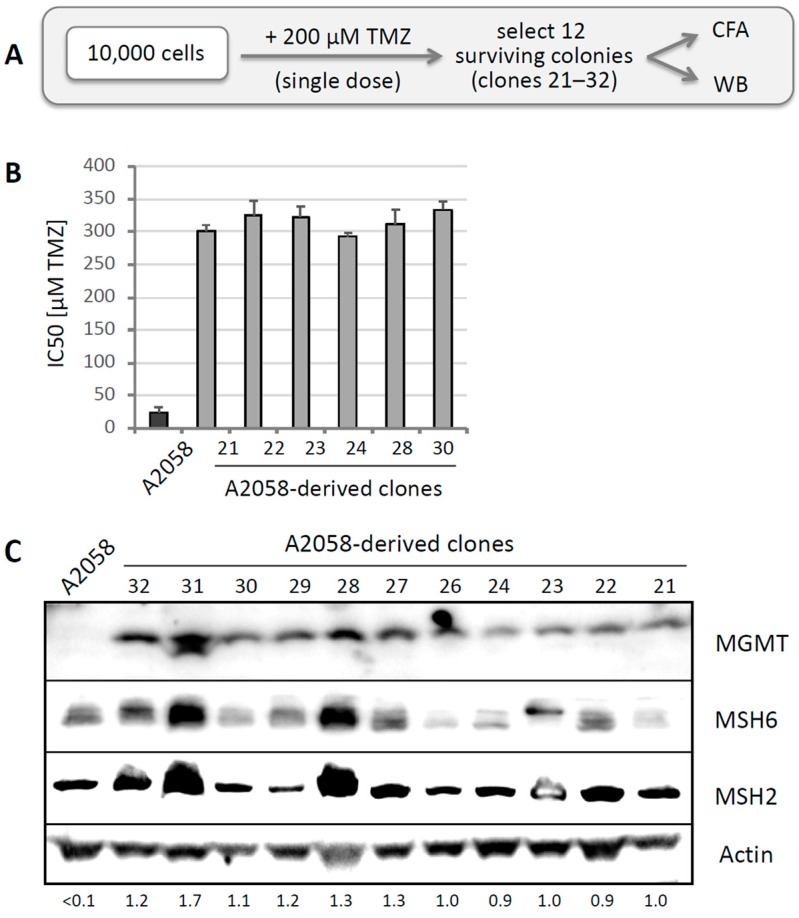
Selection of A2058 clones after high-dose TMZ treatment. (**A**) Treatment schedule was as follows: 10^5^ A2058 cells (MGMT negative) were seeded into a 10-cm dish and treated with a single dose of 200 µM TMZ for 48 h. Two weeks later, well over a thousand colonies emerged. To enable selection of well-isolated, individual clones, the colonies were pooled and re-seeded at very low density and allowed to form colonies again (in the absence of any further drug treatment). Then, 12 random colonies (numbered 21 through 32) were picked for further analysis by WB and CFA. (**B**) Six of these clones were subjected to CFA with increasing concentrations of TMZ. Shown is the average IC50 of each clone, in comparison to parental A2058 cells (left bar in black) (*n* = 2–5, ±SE). (**C**) Eleven of these cell clones were lysed, and MGMT protein levels were analyzed by WB with actin as the loading control. Left lane shows lysate from parental A2058 cells. Numbers under the blot indicate quantification of MGMT bands, with reference to the actin signal, and relative to Clone 21 (right lane, set at 1.0); <0.1 indicates below detection limit.

A similar approach was performed with MGMT-negative A2058 cells. Ten thousand cells were treated with 200 µM TMZ, which killed about 98%. From the emerging survivors, 12 individual clones were isolated (Figure 3A). CFAs were performed for half of these clones, revealing significantly increased IC50s as compared to the IC50 of parental A2058 cells (Figure 3B). Intriguingly, the elevated IC50s of these clones were around 300 µM, and thus very similar to the IC50 of MGMT-positive A375 cells (and other MGMT-positive cells). Western blot analysis of 11 of these clones revealed the prominent appearance of MGMT protein in all of them (Figure 3C). We also compared expression levels of MSH6 and MSH2, two proteins of the MMR pathway, and detected highly variable expression of these two proteins. To differentiate the potential contribution of MGMT, MSH6, and MSH2 to TMZ resistance in these clones, we performed CFAs in the presence of O6BG in clones 21, 23, 28, and 30. In these cases, inclusion of O6BG restored full sensitivity to TMZ, i.e., the IC50 was reduced from about 300 µM to ~25 µM, which is the IC50 of the MGMT-negative A2058 parental population. We therefore concluded that rapid emergence of drug resistance after single TMZ treatment was based on the presence of MGMT in these clones.

It is noteworthy that several clones, in particular clones 28 and 31, displayed prominent upregulation of MSH2 and MSH6 expression. This was interesting because increased expression of MSH6 has recently been shown to be associated with TMZ resistance [47]. However, in the case of Clone 28, our above analysis did not support a critical role for MSH6, because the addition of O6BG (i.e., inhibition of MGMT) restored full sensitivity to TMZ, assigning the drug-resistance mechanism in this clone entirely to MGMT. Nonetheless, since we did not investigate all of the other clones, there remains a possibility that alterations in MSH2 or MSH6 might contribute to the development of TMZ resistance as well.

### 3.3. TMZ Resistance Emerges after Single Drug Treatment Even at Low Concentration

**Figure 4:**
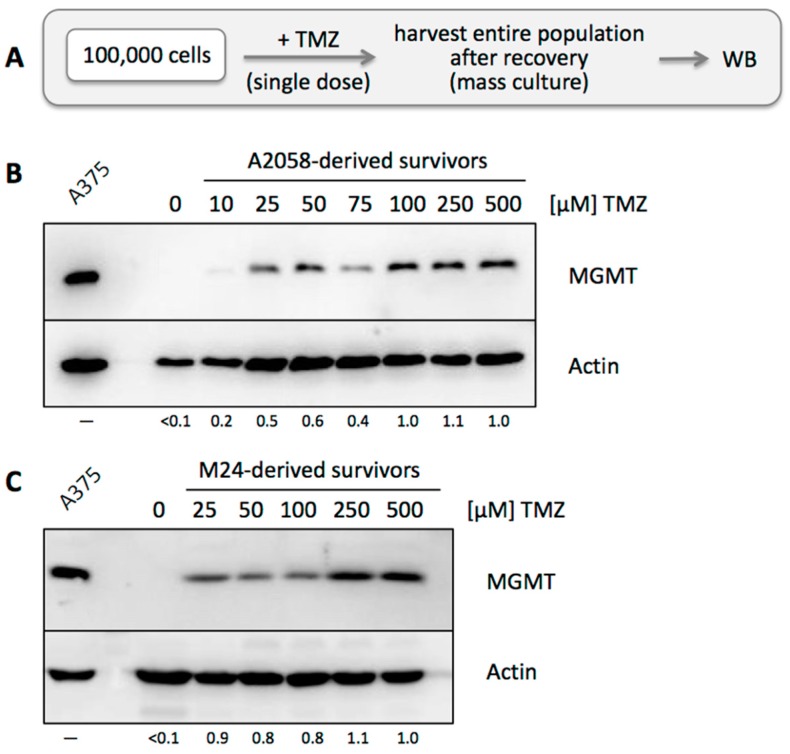
MGMT protein levels in mass cultures after TMZ treatment. (**A**) Treatment schedule was as follows: 10^5^ cells were seeded into the wells of a 6-well dish and treated with a single dose of TMZ at increasing concentrations from 0 to 500 µM for 48 h. Surviving cells were kept in culture until the population had regrown sufficiently to harvest enough cells for WB analysis. (**B**) Cell lysates were prepared from mass cultures of surviving A2058 cells and analyzed by WB for MGMT protein levels. Actin was used as a loading control. A lysate of A375 cells was used as a positive control for MGMT protein. (**C**) The same experiment was also performed with M24 cells. Please note that MGMT protein was undetectable before TMZ treatment in both cell lines. Numbers under the blots indicate quantification of MGMT bands, with reference to the actin signal, and relative to the highest TMZ concentration used (right lane, set at 1.0); <0.1 indicates below detection limit.

To complement our analysis of individual surviving clones, we next studied mass cultures of surviving cells after single TMZ treatment. MGMT-negative A2058 or M24 cells were exposed to TMZ at increasing concentrations from 10 to 500 µM (Figure 4A). After this one-time drug exposure, cells were allowed to recover. Surviving cells received fresh medium on a regular basis, until the entire population reached confluency and could be harvested for Western blot analysis. This process took longer for cell populations exposed to higher TMZ dosages, because of more extensive cell death following drug treatment. As shown in Figure 4B,C, all drug-treated cell populations had converted to MGMT-positive status. While 10 µM TMZ exerted weak effects, cells treated with only 25 µM TMZ displayed pronounced MGMT positivity that was only slightly further increased at much higher TMZ concentrations. Together, these results demonstrate rapid emergence of MGMT-positive cell populations following a single TMZ treatment, including at low, physiologically relevant concentrations.

### 3.4. MGMT-Positive Cells Exist within an MGMT-Negative Population

**Figure 5:**
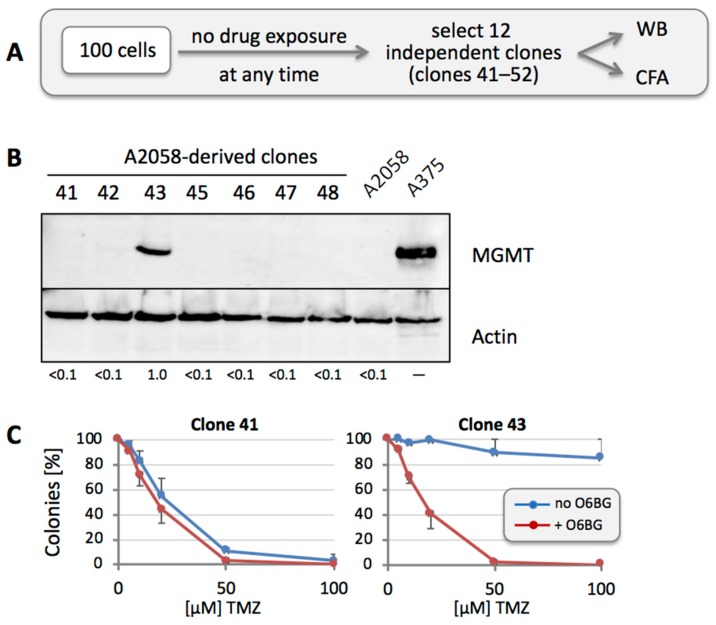
MGMT protein levels in clones derived from untreated A2058 cells. (**A**) Treatment schedule was as follows: 100 A2058 cells were seeded into a 10-cm dish. After colonies had formed (in the absence of any drug treatment), 12 of them (numbered 41 through 52) were isolated and expanded for further analysis by WB and CFA. (**B**) Cell lysates were prepared from all clones and analyzed by WB for MGMT protein levels. Actin was used as a loading control. A lysate of A375 cells was used as a positive control for MGMT protein, and a lysate of parental A2058 cells was used as the negative control. Except for clone 43, all clones were negative for MGMT protein (clones 49 to 52 were negative as well, but not included here; clone 44 was lost). Numbers under the blot indicate quantification of MGMT bands, with reference to the actin signal, and relative to Clone 43 (set at 1.0); <0.1 indicates below detection limit. (**C**) To confirm drug sensitivity in correlation with MGMT levels, clone 41 and 43 were treated with increasing concentrations of TMZ in the presence or absence of O6BG, and CFA was performed 12 days later (*n* = 3, ±SE).

We hypothesized that rapid appearance of MGMT positivity might be due to the presence of a small number of MGMT-positive cells among the otherwise MGMT-negative population of cells. To investigate this model, we randomly isolated 12 clones of individual A2058 cells in the absence of any drug treatment (Figure 5A). Western blot analysis of these cells demonstrated prominent expression of MGMT protein in one of these 12 clones (7 of the 12 clones are shown in Figure 5B). We then performed CFA with the one positive clone (Clone 43) in comparison to one of the other negative clones (Clone 41). As shown in Figure 5C, Clone 43 was highly TMZ-resistant, and inclusion of O6BG restored its drug sensitivity. In comparison, Clone 41 was TMZ sensitive (similar to the parental A2058 cell line), and inclusion of O6BG did not result in an obvious difference. Thus, this approach indicated the presence of MGMT-positive, TMZ-resistant cells among the greater MGMT-negative overall population, even before any drug treatment was applied.

**Figure 6:**
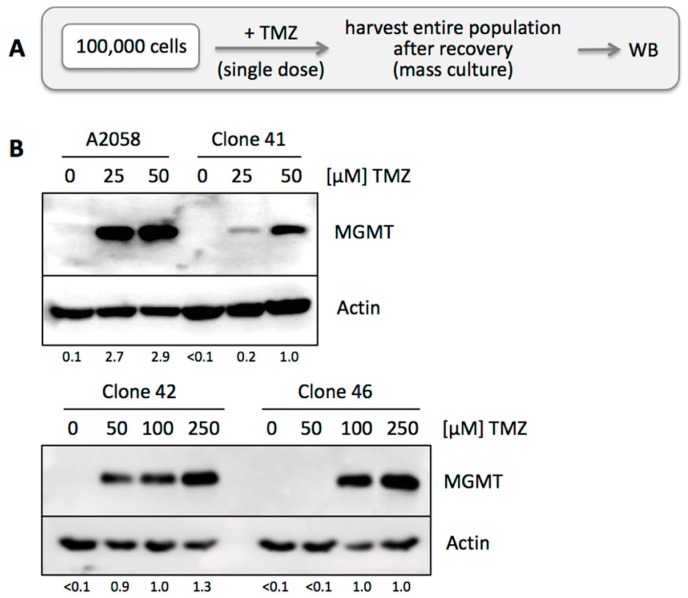
Emergence of MGMT protein in MGMT-negative clone. (**A**) Treatment schedule was as follows: 10^5^ cells were seeded into the wells of a 6-well dish and treated with a single dose of TMZ at increasing concentrations from 0 to 50 µM for 48 h. Surviving cells were kept in culture until the population had regrown sufficiently to harvest enough cells for WB analysis. (**B**) Cell lysates were prepared from mass cultures of surviving A2058 cells and from clone 41, followed by WB analysis of MGMT protein levels. Actin was used as a loading control. Numbers under the blots indicate quantification of MGMT bands, with reference to the actin signal, and relative to the intensity in the right lane (set at 1.0); <0.1 indicates below detection limit.

We next investigated the issue of stability of a given (MGMT-negative) phenotype of individual cells within the entire population, i.e., do MGMT-negative cells maintain their negative phenotype, or is there potential conversion from negative to positive status, which of course would greatly bolster the development of TMZ resistance. To address this question, we selected three of the above-described MGMT-negative clones (41, 42 and 46) and exposed each one to a single dose of TMZ at different concentrations. Cells were allowed to recover and proliferate until sufficient cells were available for harvest (Figure 6A). Western blot analysis revealed that these TMZ-treated cell populations had become MGMT positive (Figure 6B), clearly demonstrating that MGMT positivity can rapidly emerge from a cell population derived from an initially MGMT-negative clone.

### 3.5. MGMT Expression Is Not Regulated by Promoter Methylation

**Figure 7:**
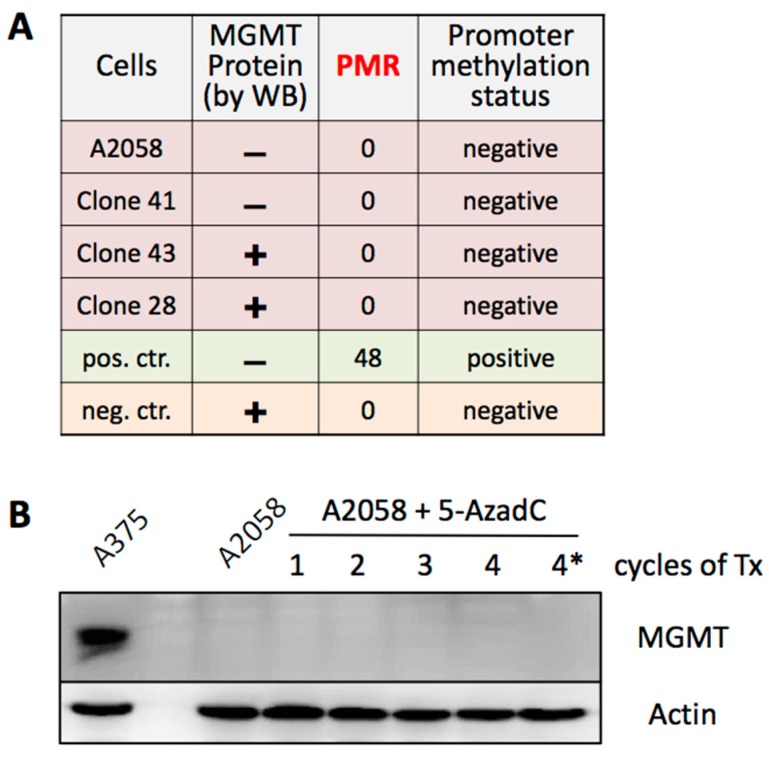
MGMT promoter methylation status. (**A**) DNA was isolated from A2058 cells and several derived clones, and analyzed for methylation status of the MGMT promoter. Reference DNA from RKO and HCT8 colorectal carcinoma cell lines was included as negative (neg. ctr.) and positive controls (pos. ctr.), respectively. PMR: percentage of methylated reference. (**B**) A2058 cells were treated (Tx) with 1, 2, 3, or 4 cycles of 5-AzaC. Thereafter, cell lysates were prepared and analyzed for MGMT protein levels by WB. Actin was used as the loading control. Cell lysate labeled 4* was from cells that were cultured for an additional 4 weeks after 4 cycles of 5-AzaC treatment.

To gain insight into the mechanism by which these melanoma cells might switch their MGMT status from negative to positive, we investigated control of promoter activity by methylation. It is well recognized that cells are able to silence MGMT expression through methylation of their MGMT promoter [18]. In fact, in the case of glioblastoma (GBM) patients, standard of care generally involves analysis of MGMT promoter methylation status in resected tumor tissue as the key predictive factor of TMZ efficacy [48]. We used MGMT-negative A2058 and Clone 41 cells, and MGMT-positive Clone 28 and Clone 43 cells, extracted DNA, and analyzed MGMT promoter methylation by MethyLight assay. However, no promoter methylation could be detected in any of the four samples (Figure 7A), indicating that differential MGMT expression in these cells was not due to differences in promoter methylation. As this finding was somewhat unexpected, it was validated by treating A2058 cells with 5-aza-deoxycytidine (5AzadC). 5AzadC is an epigenetic modifier that can be used to remove methyl groups from DNA, resulting in re-activation of promoters silenced by methylation [49]. We exposed A2058 cells to several rounds of treatment with 5AzadC, followed by Western blot analysis of MGMT expression. As shown in Figure 7B, there was no emergence of MGMT protein expression in response to extensive 5AzadC treatment. Combined, these results exclude promoter methylation as a decisive factor of differential MGMT expression in the analyzed cell lines.

### 3.6. Other Resistance Mechanisms Are Present, but Are Considerably Rarer

**Figure 8:**
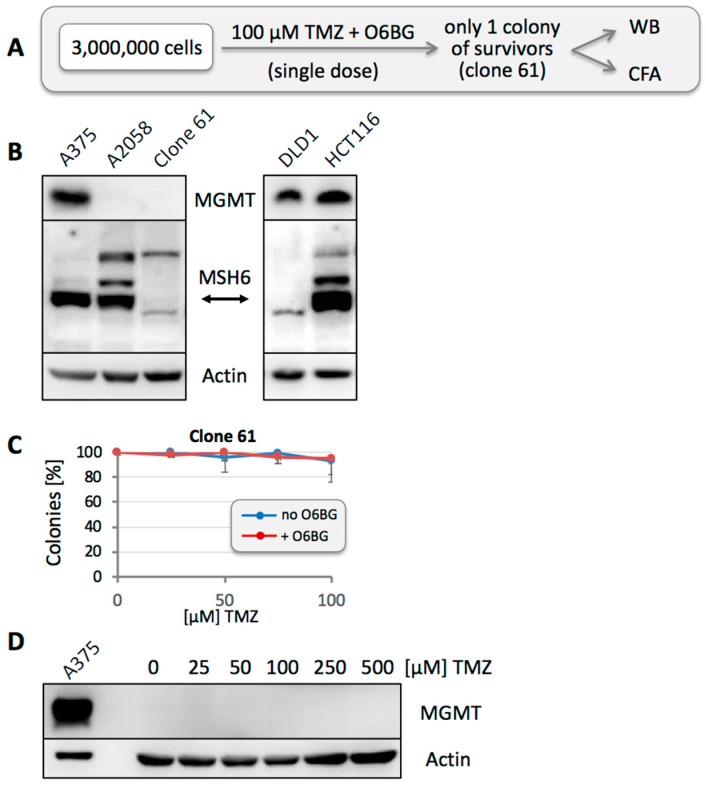
Drug resistance after high-dose TMZ plus O6BG treatment. (**A**) Treatment schedule was as follows: 3 × 10^6^ A2058 cells were seeded into a 25-cm plate and treated with 100 µM TMZ in the presence of O6BG. After about 6 weeks, one colony of surviving cells (called clone 61) emerged and was cultured for further analysis by WB and CFA. (**B**) Cell lysate was prepared from clone 61 and analyzed by WB for MGMT and MSH6 protein levels. Lysates from A375 and A2058 cells were included as MGMT-positive and -negative controls, respectively; lysates from HCT116 and DLD1 cells were included as MSH6-positive and -negative controls, respectively. (**C**) To confirm drug sensitivity in correlation with MMR deficiency, clone 61 was treated with increasing concentrations of TMZ in the presence or absence of O6BG, and CFA was performed 12 days later (*n* = 3, ±SE). (**D**) Clone 61 was treated with increasing concentrations of TMZ (single dose, as per schedule listed in Figure 4). After complete recovery, mass cultures were analyzed for MGMT protein levels by WB analysis with actin as the loading control.

While the above results characterized MGMT as a prominent contributor to TMZ resistance of these melanoma cell lines, we next asked whether other mechanisms of resistance were present as well. To investigate this aspect, we used O6BG to block any MGMT activity, thus allowing potential other elements of resistance to emerge. However, when we seeded 100,000 A2058 cells and treated them with 100 µM TMZ in the presence of O6BG, we obtained no survivors—as compared to more than a thousand surviving colonies after treatment with TMZ in the absence of O6BG (see also Figure 3). This outcome indicated that resistance based on MGMT was by far the most prevalent mechanism of resistance in these cells, and any other mechanism, if present, would have a frequency below 10^−5^. It therefore appeared necessary to investigate a larger number of cells. We seeded 3 × 10^6^ A2058 cells, followed by a one-time treatment with 100 µM TMZ in the continuous presence of O6BG (Figure 8A). After 6 weeks, a single colony of slow-growing cells emerged (Clone 61), which was expanded for further characterization. Western blot analysis revealed that these cells did not express MGMT protein, similar to the parental A2058 culture. However, unlike the A2058 culture, Clone 61 had lost expression of MSH6 (Figure 8B), and loss of this key MMR protein provided a reasonable explanation for their MGMT-independent resistance to TMZ.

Clone 61 was also used in CFAs with increasing concentrations of TMZ in the presence or absence of O6BG. As expected, the cells were highly resistant to TMZ, and inclusion of O6BG made no difference (Figure 8C), confirming that MGMT did not contribute to these cells’ resistance phenotype. Furthermore, similar to the treatment approach presented in Figure 4, we seeded 100,000 Clone 61 cells per well and treated these populations with increasing concentrations of TMZ, followed by Western blot analysis after cells had recovered from drug treatment. Unlike the results obtained with parental A2058 cells (Figure 4) and other selected clones (Figure 6), TMZ treatment of Clone 61 did not result in the emergence of MGMT-positive populations (Figure 8D). On one hand, this outcome suggested that loss of MSH6 was sufficient for drug resistance, and MGMT was not required. On the other hand, in view of the very rapid emergence of MGMT positivity after treatment of A2058 or M24 cells with TMZ (Figure 4), it also diminished the possibility that TMZ in general might trigger stable MGMT expression (as opposed to the selection of pre-existing MGMT-positive cells).

### 3.7. Repeated TMZ Treatment Accelerates the Emergence of Resistance

**Figure 9:**
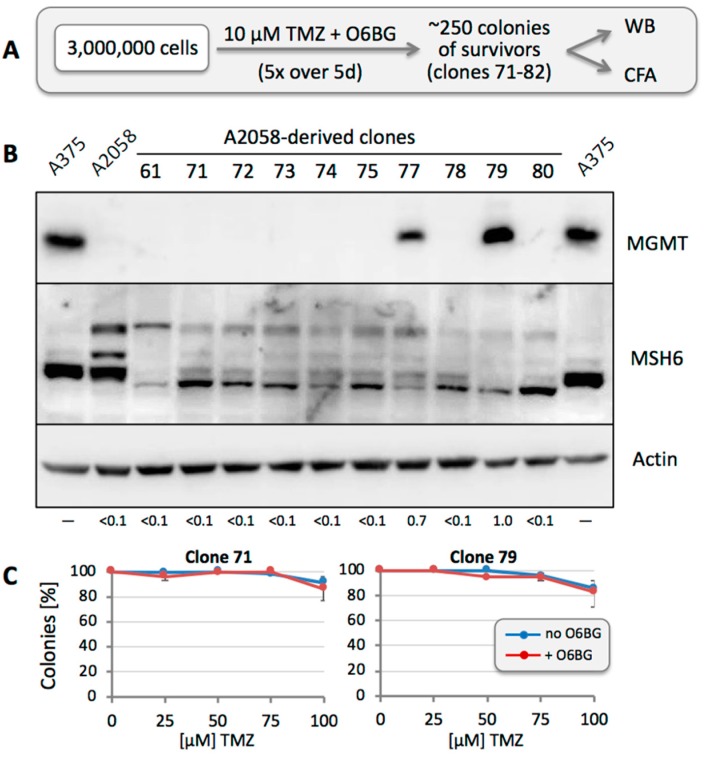
Drug resistance after repeated low-dose TMZ plus O6BG treatment. (**A**) Treatment schedule was as follows: 3 × 10^6^ A2058 cells were seeded into a 25-cm plate and treated with 10 µM TMZ in the presence of O6BG once daily over 5 days. After about 15 days, there were approximately 250 colonies, which were pooled and expanded. After sufficient proliferation, 3 × 10^6^ of these cells were seeded again into a 25-cm plate and exposed to a single treatment of 100 µM TMZ in the presence of O6BG. Another 15 days later, approximately 2000 colonies emerged, which were sub-cultured to allow for the isolation of 12 individual clones (numbered 71 through 82), which were analyzed by WB and CFA. (**B**) Cell lysates were prepared from all clones and analyzed by WB for MGMT and MSH6 protein levels. Lysates from A375 and A2058 cells were included for comparison. (Clones 81 and 82 are not included in this figure; clone 76 was lost.) Numbers under the blot indicate quantification of MGMT bands, with reference to the actin signal, and relative to Clone 79 (set at 1.0); <0.1 indicates below detection limit. (**C**) To confirm drug sensitivity in correlation with MGMT or MMR deficiency, clones 71 and 79 were treated with increasing concentrations of TMZ in the presence or absence of O6BG, and CFA was performed 12 days later (*n* = 3, ±SE).

In all of the above experiments, TMZ was applied as a single treatment. In clinical use, however, it is given repeatedly. For instance, during the adjuvant phase after completion of the radiation schedule, it usually is given for 5 consecutive days every 4 weeks [50]. We therefore pursued the question as to whether repeated drug treatments at physiologically relevant concentrations would increase the likelihood of resistance development. Because the pre-existing presence of MGMT-positive cells was expected to quickly dominate the cell population in response to TMZ treatment, we included O6BG in these experiments in order to eliminate protection by MGMT. Three million cells were seeded and treated with 10 µM TMZ in the continuous presence of O6BG once daily for 5 consecutive days. This resulted in about 250 colonies of surviving cells (Figure 9A).

We isolated 12 of these clones and performed Western blot analysis, which revealed loss of MSH6 in all cases (Figure 9B). Intriguingly, two of the clones (77 and 79) also were MGMT positive. CFAs of several of these clones confirmed that they were highly TMZ-resistant, and inclusion of O6BG had no effect (see representative examples in Figure 9C). It is noteworthy that O6BG also had no effect in those 2 MGMT-positive clones, revealing that their resistance status was not determined by MGMT, but that the presence of MGMT was probably of stochastic origin, as described above. Altogether, these results present loss of MSH6 expression as a protective backup mechanism of tumor cells under conditions of inactivated MGMT.

### 3.8. Emergence of MGMT-Driven TMZ Resistance Occurs In Vivo 

**Figure 10:**
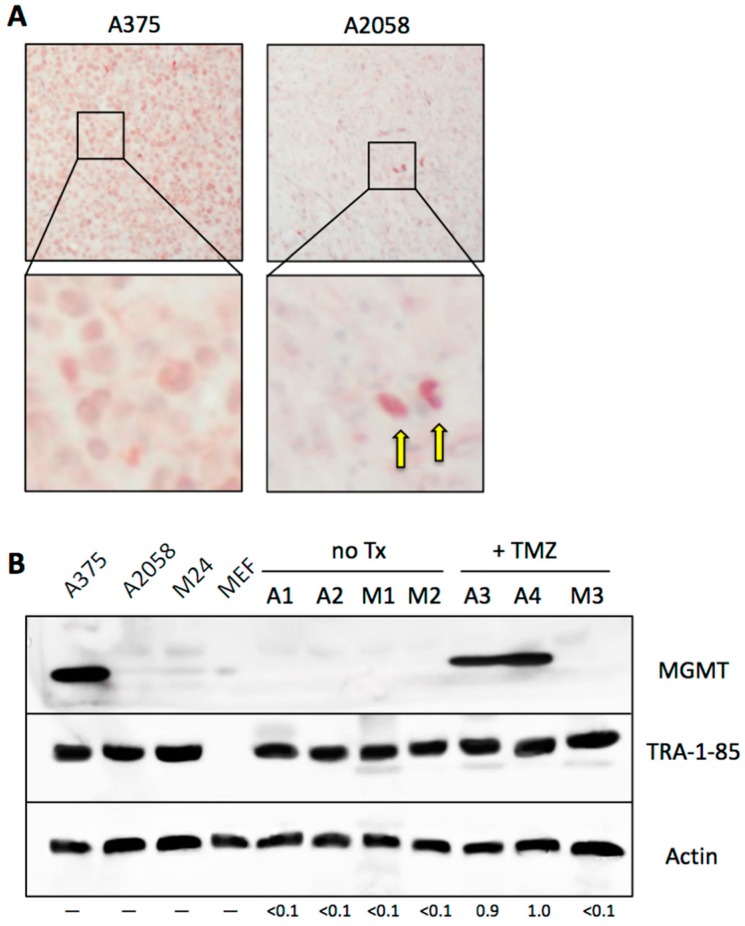
MGMT expression in tumor tissue. (**A**) Nude mice were injected subcutaneously with 4 × 10^6^ MGMT-positive A375 cells or with MGMT-negative A2058 cells. Once pea-sized tumors had developed, tumor tissue was harvested and subjected to immunostaining for human MGMT. While A375 tissue showed positive staining in most cells, A2058 tissue revealed positivity only in a small minority of cells (2 examples are indicated by arrows in the enlarged section). (**B**) Western blot was performed with crude lysates from cells cultured in vitro (A375, A2058, M24, and MEF) and from xenograft tumor tissues (A1–A4: tumors derived from implanted A2058 cells; M1–M3: tumors derived from implanted M24 cells), using a human-specific MGMT antibody. A1, A2, M1 and M2 are tumors from mice that only received vehicle treatment (control); A3, A4 and M3 are tumors from mice that received TMZ therapy (M4 animal was lost). To confirm the presence of human tumor cells in tissues harvested from mice—especially in those lanes where the MGMT signal was negative—we used an antibody against TRA-1-85/CD147, an epitope that is present only on human cells, but not mouse cells. Please note that specificity of this antibody was confirmed by lack of reactivity with the lane containing lysate from mouse embryo fibroblasts (MEF). Actin was used as the loading control. Numbers under the blot indicate quantification of MGMT bands, with reference to the actin signal, and relative to A4 sample (set at 1.0); <0.1 indicates below detection limit; only lanes with tumor tissue samples were measured.

Finally, we investigated MGMT expression and its relation to TMZ resistance under in vivo conditions. Mice were implanted with MGMT-positive A375 or MGMT-negative A2058 cells. In the absence of any drug treatment, well-grown tumors were collected after three weeks and subjected to immunohistochemistry with a human-specific MGMT antibody. As shown in Figure 10A, tissue grown from A375 cells stained positive for MGMT, as expected. In comparison, tissue derived from A2058 cells appeared negative, although a small number of positive cells could clearly be recognized, consistent with our in vitro finding that the A2058 cell population harbors a minority of MGMT-positive members.

We then asked the question whether in vivo treatment with TMZ would trigger the selection and accumulation of MGMT-positive cells, as it did in vitro. To establish proof-of-principle of this process, we implanted A2058 and M24 cells into the flanks of mice. Once palpable tumors had developed, half the mice received daily TMZ treatment for 2 weeks, while the other half received vehicle only. At the end of the treatment period, vehicle-treated animals presented with large tumors, which necessitated euthanasia, along with collection of tumor tissue. At this time point, TMZ-treated animals had only very small tumors, indicating clear therapeutic activity of TMZ. These latter animals were not euthanized, but were kept for another 3 weeks without any further TMZ treatment. During these 3 weeks, tumor growth resumed, and the tissues were collected at the end of this time period.

Figure 10B presents Western blot analysis of tumor tissues from this experiment. A1–A4 represent tumor tissues derived from A2058 cells, whereas M1–M3 are from M24 tissues. As shown, tumor tissues from vehicle-treated animals (A1, A2, M1, M2) are MGMT-negative, just like their in vitro-cultured counterparts (shown on the left side of the blot). In contrast, both A2058 tumors from TMZ-treated animals (A3, A4) were strongly MGMT-positive. In comparison, the one tumor obtained from M24 cells in TMZ-treated animals was MGMT-negative. In conclusion, in the case of A2058, we could establish proof-of-principle that TMZ treatment triggered the selection of MGMT-positive cells, which were derived from a pre-existing minority of cells that survived and eventually dominated the tumor mass.

## 4. Discussion

Methylation of the O6-position of guanine constitutes the key toxic lesion placed onto the DNA strand by TMZ. In cells expressing MGMT, the DNA repair mechanism of this protein removes this foreign methyl group, thus eliminating the toxic threat and protecting survival of the cells. However, in the absence of MGMT, O6-methylated guanine mispairs with thymidine, triggering futile cycles of attempted DNA repair by the MMR system, resulting in DNA strand breaks and apoptosis. Intriguingly, if the MMR system is defective, as is the case when key MMR proteins are absent, O6-methylguanine: thymidine mispairs do not trigger cell death; rather, the cells survive at the expense of an increased rate of mutations, in particular G:C to A:T transitions. For this reason, MMR-deficient cells, for example those lacking expression of the MMR protein MSH6, are profoundly resistant to TMZ [12,51,52,53].

The key findings of our study are as follows: (i) two established melanoma cell lines, which appear to lack MGMT expression, in fact harbor a small percentage of MGMT-positive cells. (ii) MGMT expression is stochastic, and isolation of individual, MGMT-negative clones again results in a population with mixed MGMT status. (iii) A one-time exposure to TMZ, at physiologically relevant concentrations, effectively depletes the MGMT-negative population and promptly enriches for MGMT-positive, TMZ-resistant survivors. (iv) The combination of TMZ with O6BG prevents the emergence of MGMT-based TMZ resistance; however, an alternative resistance mechanism, loss of the MMR protein MSH6, appears (although at much lower frequency).

Our study focused on TMZ concentrations that are physiologically relevant. Peak concentrations of TMZ measured in the plasma of melanoma patients are about 50 µM [45,46]. Based on these levels, we considered cells that survived treatment with >50 µM TMZ as unresponsive to TMZ. Intriguingly, while the IC50s of our two MGMT-negative cell lines were in the range of 10–20 µM, thus marking them as TMZ sensitive, we nonetheless obtained a few surviving colonies even at 100 µM TMZ (and above). In the case of A2058, this TMZ-resistant fraction was 1–3%, whereas with M24 cells it was 0.5–1.0%. The addition of O6BG substantially reduced colony survival at these elevated TMZ concentrations, providing an early indication that MGMT was involved in conferring drug resistance (Figure 1).

To further investigate the mechanisms of TMZ resistance in these cells, we applied an in vitro strategy that used only a single, one-time drug exposure. We hypothesized that any survival of cells under such conditions must be due to a pre-existing resistance mechanism, quite likely based on the involvement of MGMT. The veracity of this model was supported by the following findings: (i) When MGMT-negative A2058 or M24 cells were exposed to TMZ, the resulting surviving cell population had become strongly MGMT positive (Figure 4). Because only a single exposure to TMZ had been applied, the resistance mechanism must have been already present at the time of addition of the drug. (ii) When we isolated 12 clones, picked at random from A2058 cells in the absence of any drug treatment, one of them turned out to be strongly MGMT-positive (Clone 43) (Figure 5), indicating that rare MGMT-positive cells were present among the general MGMT-negative cell population, even before any TMZ treatment was applied. This was further substantiated by an independent experiment, where two of 12 individually isolated clones after treatment of cells with TMZ + O6BG turned out to be strongly MGMT-positive, even though MGMT was not involved in the drug resistance mechanism of these (MSH6-deficient) cells (Figure 9).

On one hand, it makes sense that the presence of even a few MGMT-positive cells among an otherwise MGMT-negative population will result in rapid expansion of this minority fraction upon TMZ treatment. On the other hand, it was unclear whether MGMT status within such a population was stable or fluid. We therefore addressed the question whether MGMT-negative cells among a mixed population would retain their negative status, or whether they would be able to convert and produce progeny with mixed MGMT status. Two experimental results indicated that the latter model applied: (i) when individual A2058 cells were isolated and cloned, the majority of them presented as MGMT-negative in Western blot analysis (Figure 5); however, upon single treatment with TMZ, all these cell cultures promptly converted to strong MGMT-positive status (Figure 6), in a fashion similar to the parental A2058 population (Figure 4). (ii) However, extensive TMZ treatment of Clone 61, which represents A2058 cells with TMZ resistance based on MMR deficiency, did not result in increased MGMT expression (Figure 8). This outcome constituted an important control, because it excluded the possibility that mere treatment with TMZ might have been the trigger for increased MGMT expression in MGMT-negative clones. Lack of MGMT stimulation by TMZ is further supported by a study showing that TMZ exposure of melanoma cells in vitro does not alter MGMT mRNA expression [54].

Combined, the above results favor a model where MGMT expression stochastically appears in a small fraction of the melanoma cell population, thus providing the seeds of TMZ resistance even before the commencement of any TMZ treatment. The underlying mechanism of this switch between different MGMT statuses remains to be established, although we excluded differential methylation of the MGMT promoter as an epigenetic control of this event (Figure 7). We focused on promoter methylation, because in glioblastoma this modification has been recognized as a predictive and prognostic factor for therapy with methylating agents, such as TMZ, and determination of MGMT promoter methylation status is standard practice for this tumor type [55,56]. In melanoma, however, the role of this epigenetic modification is less clear. The percentage of tumors with methylated MGMT is lower than in glioblastoma, and clinical studies with melanoma patients concluded that MGMT promoter methylation status did not correlate with clinical outcome after TMZ [57,58]. The results shown in Figure 7 are in agreement with the view that promoter methylation does not appear to play a critical role in melanoma. Our analysis of A2058 cells and derived Clone 41 reveals that their lack of MGMT protein expression (Figure 5) is not due to silencing of the promoter by methylation (Figure 7); as a corollary, the rare emergence of MGMT-positive cells, such as Clone 43 (Figure 5), from an otherwise MGMT-negative cell population cannot be caused by promoter demethylation. In any case, irrespective of the underlying control mechanism, our study provides proof of principle of stochastic MGMT expression in vitro, and there are indications that similar events might take place in patient tissue. For example, a few studies have documented intratumoral heterogeneity with regard to MGMT status in patient samples derived from melanoma [59,60], as well as glioblastoma [61].

In the clinical setting, efforts were undertaken to overwhelm the well-recognized MGMT obstacle via addition of MGMT inhibitors to TMZ therapy. However, despite the potent activity of such combinations in pre-clinical models [23,35,36], neither glioblastoma nor melanoma patients experienced significant benefit over TMZ alone [33,34,62,63] and the underlying reasons have remained unclear. Consistent with earlier in vitro studies, we found that TMZ combined with O6BG was hugely potent in all our melanoma cell lines, irrespective of their MGMT status. For example, in MGMT-negative A20158 cells, where a single treatment with 100 µM TMZ killed 98% of the cells, inclusion of O6BG increased efficacy by 10,000-fold to 99.9999% (Figure 8). Similarly, in M24 cells, O6BG increased TMZ efficacy by a factor of >2000. In the face of such enormous enhancements in vitro, it is puzzling why they cannot be translated into the clinic—although our analysis of surviving clones might provide some basis for rational speculation (below).

In comparison to single treatment with 100 µM TMZ + O6BG, where we obtained only 1 surviving clone (Figure 8), we obtained >100-fold more survivors when we applied conditions that were more clinically relevant, namely a 5-day cycle of 10 µM TMZ + O6BG. Overall cell killing was still very high at about 99.99% (Figure 9). As expected, MGMT did not play a role in TMZ resistance of these survivors; instead, 12 out of 12 analyzed clones revealed prominent down-regulation, if not loss, of MSH6 expression, a key component of the MMR pathway. Loss of function of MSH6 (or of other MMR proteins) has been shown to confer profound resistance to TMZ in a variety of tumor cell lines [52]. In glioblastoma, MSH6 mutations or loss of expression have been associated with tumor progression during TMZ treatment and ensuing TMZ resistance [12,13,64], but the role of MSH6 in clinical melanoma is less well characterized [65].

Contrary to the loss of MSH6, a recent study in glioblastoma found increased expression of MSH6, which was associated with TMZ resistance [47]. The authors proposed that aberrantly increased MSH6 might disrupt fine-tuned cooperation among MMR proteins, resulting in incompetence of DNA repair and tolerance of TMZ-induced lesions. This tentative model is intriguing in view of our findings that several clones of TMZ-resistant A2058 cells displayed increased expression of MSH2 and MSH6 proteins (Figure 3C). Although among the clones we analyzed, the primary resistance mechanisms was based on MGMT, it is conceivable that in some of the other clones elevated expression of these MMR proteins might contribute to the development of TMZ resistance.

It remains to be determined how MSH6 is upregulated in some cells (Figure 3; reference [47]), but downregulated in others (Figure 8 and Figure 9). It is possible that TMZ-induced mutations may play a role. This might be particularly relevant in response to repeated drug administration, as is the standard approach in the clinic [50] and in many pre-clinical studies aimed at generating drug-resistant variants [8,9,10,11,47]. Also, our MSH6-deficient clones shown in Figure 9 were derived after 5-time TMZ treatment. On the other hand, our MSH6-deficient Clone 61 originated from a single TMZ exposure of the parental cells, and so did the MSH6-overexpressing clones shown in Figure 3, tentatively minimizing a role for mutations, but favoring more immediate drug impact or selection of pre-existing cells with aberrant MSH6 expression. In any case, irrespective of the underlying molecular mechanism, our finding that a single, clinically relevant cycle of TMZ/O6BG results in survivors with a consistent MSH6-deficient phenotype demonstrates the emergence of a secondary resistance mechanism beyond MGMT, and provides a reasonable explanation for why tumor cells might escape TMZ/O6BG treatment in the clinic.

Despite the emergence of secondary resistant cells, treatment with TMZ/O6BG in vitro still achieves 99.99% cell killing, which was observed in different preclinical studies and has provided the rationale to test this drug combination in patients. So why is a corresponding positive effect not observed in the clinic? Naturally, the preclinical conditions are different from the situation in a patient, which complicates direct comparisons and invites reasonable speculations: Our observation that TMZ/O6BG-treated cells rapidly produce an MMR-deficient phenotype is intriguing in the context of other studies showing that exposure to alkylating agents like TMZ can foster a hypermutational phenotype [12,13,14]. This model implies that, in addition to conferring TMZ resistance, impaired MMR leads to many additional mutations that provide a growth advantage and accelerate tumor progression and cross-resistance to additional drugs with different targets [7,12,13,14,66,67]. While this scenario seems detrimental for the patient, it may open new avenues for therapeutic intervention with checkpoint inhibitors, which appear to have increased efficacy when applied to genetically unstable and hypermutated tumors [68,69]. In fact, pembrolizumab, an antibody inhibitor of programmed cell death 1 (PD-1) receptor on T cells, which initially was approved to treat advanced melanoma, received additional approval for MMR-deficient solid cancers, representing the first time the FDA approved a cancer drug based on tumor genetics rather than tumor site [70,71]. Thus, while TMZ combined with O6BG does not appear to yield impressive long-term results, its short-term benefits, in addition to buying melanoma patients some extra time, could potentially prime the recurrent tumor tissue to greater sensitivity to checkpoint inhibitors, such as pembrolizumab. While this model is speculative, it is testable and should provide impetus for further studies.

## 5. Conclusions

**Figure 11:**
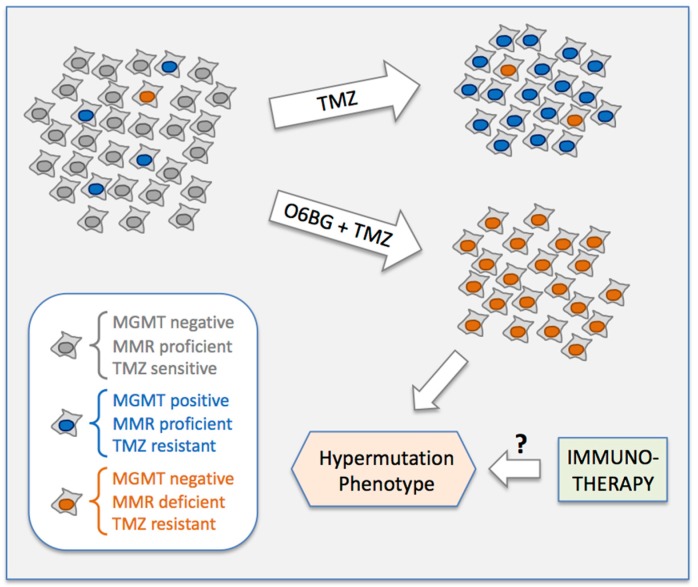
Model of selection of drug-resistant melanoma cell populations during treatment with TMZ, with or without added O6BG. Please note that rare MGMT-positive cells (blue) and even rarer MMR-deficient cells (orange) already are present in the parental cell population before the onset of drug treatment. Based on our results, there can also be mixed MGMT-positive/MMR-deficient cells (not displayed here); in such cells, MGMT would ensure survival in case of TMZ treatment, and MMR-deficiency would ensure survival in case of TMZ + O6BG combination treatment. See further details in Discussion and Conclusions sections.

Our study reveals rare expression of MGMT as a stochastically pre-existing, key determinant of TMZ resistance in melanoma cell lines (Figure 11). Upon treatment with TMZ at physiologically relevant concentrations, MGMT-negative cells are eliminated, but the tiny fraction of MGMT-positive cells survives and becomes the dominant, drug-resistant population. Combining TMZ with the MGMT inhibitor O6BG suppresses survival of MGMT-positive cells, but drug-resistant survivors still emerge, due to the presence of even rarer cells that harbor defective MMR (i.e., impaired MSH6 expression) that conveys TMZ resistance. These findings might explain, at least in part, why TMZ-based treatment regimens, with or without added O6BG, do not achieve impressive clinical outcomes in melanoma patients.

Of note, MMR-deficient cells, which escape TMZ + O6BG combination treatment, are prone to develop a hypermutation phenotype, which results in increased display of neoantigens as potential targets for the immune system. It is thus conceivable that melanoma patients recurring after TMZ + O6BG might respond favorably to interventions that administer immune checkpoint inhibitors (Figure 11).

## Abbreviations

5-AzaC	5-Aza-deoxycytidine
CFA	Colony-formation assay
MGMT	O6-methylguanine-DNA methyltransferase
MMR	Mismatch repair
O6BG	O6-Benzylguanine
TMZ	Temozolomide
WB	Western blot
